# Validation of the flow index to detect low inspiratory effort during pressure support ventilation

**DOI:** 10.1186/s13613-022-01063-z

**Published:** 2022-09-26

**Authors:** Ming-Yue Miao, Wei Chen, Yi-Min Zhou, Ran Gao, De-Jing Song, Shu-Peng Wang, Yan-Lin Yang, Linlin Zhang, Jian-Xin Zhou

**Affiliations:** 1 Department of Critical Care Medicine, Beijing Tiantan Hospital, Capital Medical University, No. 119, South 4th Ring West Road, Fengtai District, Beijing, 100070 China; 2 Department of Critical Care Medicine, Beijing Shijitan Hospital, Capital Medical University, No. 10, Tieyi Road Haidian District, Beijing, 100038 China; 3 Beijing Engineering Research Center of Digital Healthcare for Neurological Diseases, Beijing Tiantan Hospital, Capital Medical University, Beijing, China; 4 Surgical Intensive Care Unit, China-Japan Friendship Hospital, Beijing, China

**Keywords:** Mechanical ventilation, Inspiratory effort, Monitoring, Flow index, Diagnostic accuracy

## Abstract

**Background:**

Bedside assessment of low levels of inspiratory effort, which are probably insufficient to prevent muscle atrophy, is challenging. The flow index, which is derived from the analysis of the inspiratory portion of the flow–time waveform, has been recently introduced as a non-invasive parameter to evaluate the inspiratory effort. The primary objective of the present study was to provide an external validation of the flow index to detect low inspiratory effort.

**Methods:**

Datasets containing flow, airway pressure, and esophageal pressure (P_es_)–time waveforms were obtained from a previously published study in 100 acute brain-injured patients undergoing pressure support ventilation. Waveforms data were analyzed offline. A low inspiratory effort was defined by one of the following criteria, work of breathing (WOB) less than 0.3 J/L, P_es_–time product (PTP_es_) per minute less than 50 cmH_2_O•s/min, or inspiratory muscle pressure (P_mus_) less than 5 cmH_2_O, adding “or occurrence of ineffective effort more than 10%” for all criteria. The flow index was calculated according to previously reported method. The association of flow index with P_es_-derived parameters of effort was investigated. The diagnostic accuracy of the flow index to detect low effort was analyzed.

**Results:**

Moderate correlations were found between flow index and WOB, P_mus_, and PTP_es_ per breath and per minute (Pearson’s correlation coefficients ranged from 0.546 to 0.634, *P* < 0.001). The incidence of low inspiratory effort was 62%, 51%, and 55% using the definition of WOB, PTP_es_ per minute, and P_mus_, respectively. The area under the receiver operating characteristic curve for flow index to diagnose low effort was 0.88, 0.81, and 0.88, for the three respective definition. By using the cutoff value of flow index less than 2.1, the diagnostic performance for the three definitions showed sensitivity of 0.95–0.96, specificity of 0.57–0.71, positive predictive value of 0.70–0.84, and negative predictive value of 0.90–0.93.

**Conclusions:**

The flow index is associated with P_es_-based inspiratory effort measurements. Flow index can be used as a valid instrument to screen low inspiratory effort with a high probability to exclude cases without the condition.

## Background

Pressure support ventilation (PSV) is one of the most widely used modes for mechanically ventilated patients [1]. The main advantage of PSV is to provide variable inspiratory flow to match the patient’s inspiratory effort, but so far there is no consensus on the adjustment of the optimal pressure support level [2]. Recent studies suggest that low levels of inspiratory effort due to over-assistance may adversely affect the respiratory system, probably leading to diaphragm atrophy and contractile dysfunction due to disuse [3, 4]. Bedside evaluation of potential injurious low effort is challenging. Indeed, the absence of signs related to respiratory workload (respiratory distress or recruitment of accessory respiratory muscles) is not sufficient: most over-assisted patients appear calm and comfortable [5, 6]. Therefore, early detection of low effort is essential for the appropriate management of patients receiving PSV.

Numerous instruments have been designed to assess inspiratory effort [7, 8]. Up to now, measurements based on esophageal pressure (P_es_) are still being treated as the gold standard, including tidal swing of Pes (ΔP_es_), inspiratory muscle pressure (P_mus_), P_es_–time product (PTP_es_), and work of breathing (WOB) [9, 10]. However, these parameters are usually used for research purposes and not for routine clinical monitoring, mainly because they require relatively invasive procedures, special equipment, and high expertise with complex calculations. Recently, several non-invasive methods have been investigated, including airway occlusion pressure (P0.1) [11], the swing in airway pressure (P_aw_) generated by the patient’s respiratory effort against the occluded airway (ΔP_OCC_) [12], and pressure muscle index (PMI) [13], and the results show that these parameters can reliably assess inspiratory effort. In 2021, Albani and colleagues introduced a new parameter derived from the analysis of the inspiratory portion of the flow–time waveform, the flow index, which is independently correlated with inspiratory effort in patients receiving PSV [14]. Data from the same group of patients showed that the flow index could accurately identify high and low inspiratory effort [15]. The advantage of this monitoring method is that no airway manipulation is required, and continuous monitoring can be accommodated if automatic curve fitting is integrated into the ventilator design. However, the study was single-center and lacked additional evidence to verify its validity.

In the present study, we performed a secondary analysis of previously published data on brain-injured patients [16]. The primary aim was to provide external validation of the flow index to detect potential injurious low inspiratory effort. In addition, we specifically investigated whether the flow index could be used as a screening tool because of its continuous measurement characteristics.

## Methods

This was a secondary analysis of data obtained from a previously published prospective observational cohort study (ClinicalTrials.gov: NCT03212482) [16]. Anonymous use of the data was approved by the Institutional Review Board of Beijing Tiantan Hospital, Capital Medical University (KY 2017–028-02).

### Data collection

More detailed information about the previous study can be found in the original publication [16]. The study enrolled 100 acute brain-injured patients undergoing mechanical ventilation (AVEA ventilator, CareFusion Co., USA) and P_es_ monitoring (SmartCath-G catheter, CareFusion Co., San Diego, CA, USA). The position of the esophageal balloon was confirmed by an occlusion test [17]. Flow, P_aw_, and P_es_ waveforms were recorded at 100 Hz for 15 min using the ventilator acquisition system (VOXP Research Data Collector 3.2, Applied Biosignals GmbH, Weener, Germany). The settings of the ventilator remained unchanged during the 15-min recording period. At the end of the recording, an arterial blood gas analysis was performed.

In the study unit, the ventilator mode was usually changed to PSV when all ventilator breaths were triggered by the patient during assist/control ventilation. Pressure support was set to obtain tidal volume (V_T_) of 6–8 ml/kg predicted body weight (PBW) with the respiratory rate (RR) lower than 30 breaths/min, and to maintain an arterial partial pressure of carbon dioxide of 35–40 mmHg (usually performed twice daily) as long as possible [16]. The trigger sensitivity was usually set as 1–2 L/min for the flow-trigger and 1.5–3 cmH_2_O for the pressure-trigger. The inspiratory-to-expiratory cycling was usually set as 25–30% of peak inspiratory flow. Inspired oxygen fraction (FiO_2_) and positive end-expiratory pressure (PEEP) were set according to the oxygenation condition of the patient. The first dataset undergoing PSV in each patient was selected for the present analysis. We chose five consecutive stable breaths without P_es_ artifacts, swallowing, and patient–ventilator asynchrony from the last 5 min in each 15-min dataset, and measurements were averaged. Data were offline analyzed by using a dedicated software (ICU-Lab 2.5 software package, KleisTEK, Bari, Italy).

### Definitions and measurements

Measurements of respiratory mechanics variables were in accordance with previous recommendations [9, 10, 14]. The onset of inspiratory effort was defined as the point of negative deflection of P_es_ with a rapid change in slope [18]. The onset and the end of ventilator insufflation were identified as the first and the last positive value in the flow–time recording, respectively [10].

Inspiratory V_T_ was integrated using a flow–time waveform. RR was calculated as 60 s divided by the time of the total breathing cycle (s) in each measured breath and averaged over the five selected breaths. The rapid shallow breathing index (RSBI) was calculated as the ratio between RR (breaths/min) and V_T_ (L) [19]. Minute ventilation (MV) was also calculated.

The inspiratory ∆P_es_ was calculated as the difference in P_es_ between the onset of inspiratory effort and the maximal negative deflection during inspiration. Intrinsic PEEP (PEEPi) was measured as the P_es_ dropping from the onset of inspiratory effort to the onset of ventilator insufflation. P_mus_ was calculated as the maximal difference between the static recoil pressure of the chest wall (P_cw_) and P_es_ during inspiration. A theoretical value of chest wall compliance, estimated as 4% of the predicted value of vital capacity [20], was used to construct P_cw_.

The PTP_es_ per breath (cmH2O•s) was measured as the area subtended by the P_es_–time and P_cw_–time curve from the onset of inspiratory effort to the end of ventilator insufflation [10]. PTP_es_ per minute was calculated as the product of PTP_es_ and RR, which was expressed as cmH2O•s/min. The inspiratory WOB was measured using the Campbell diagram and was expressed as joules/liter (J/L) [10, 21].

Original results of ineffective triggering were used in the present study [16]. The ineffective effort (IE) index was calculated as the percentage of ineffective triggers in total breaths in the entire 15-min dataset [22].

**Fig. 1:**
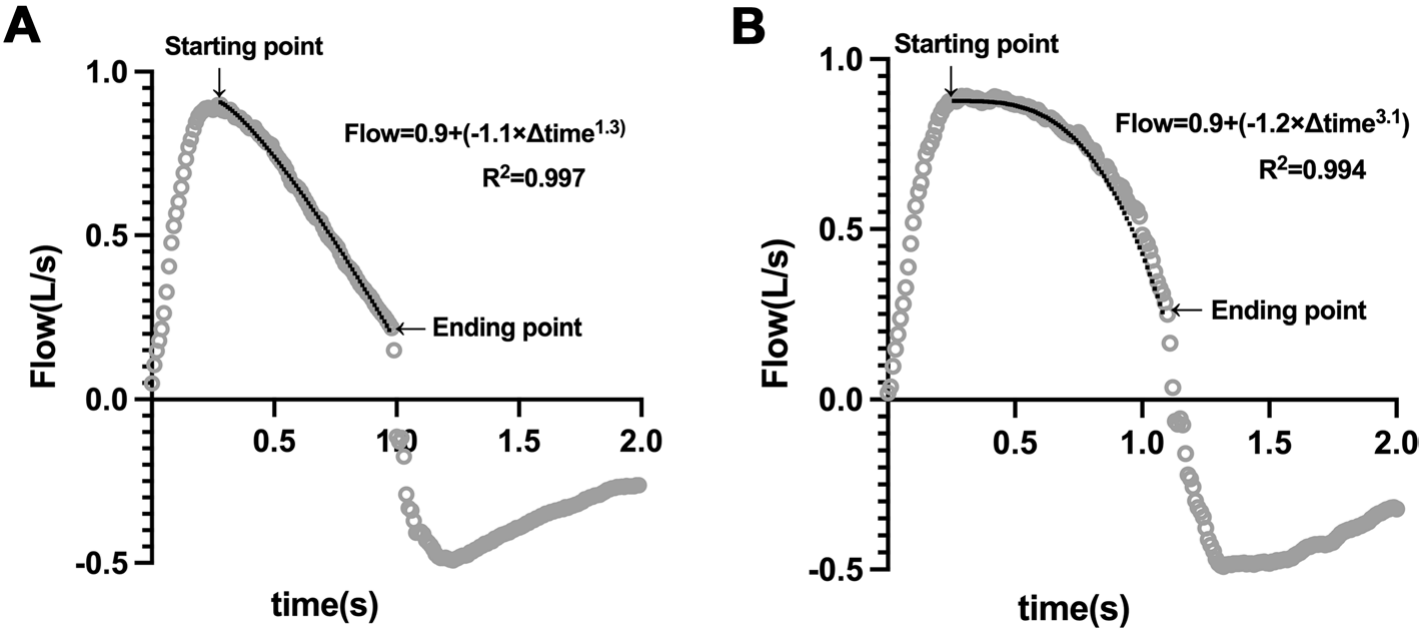
Schematic show of flow index measurement. Flow–time waveform (grey circle, 100 Hz) for a single breath under pressure support ventilation is shown. The starting point of flow–time fitting was defined as the flow increased less than 1% of the preceding measurement, and the ending point was identified as the flow decreased more than 10% of the former measurement. The equation of fitting is also shown. The solid black line indicates the flow–time fitting curve (*R*^2^ = 0.997 and 0.994). The flow index was 1.3 and 3.1 in panel A and panel B, respectively

The flow index was calculated according to the method introduced by Albani and coworkers based on flow–time curve analysis during PSV [14]. The starting point on the flow–time waveform during inspiratory was defined as the flow increased less than 1% of the preceding measurement, and the ending point was identified as the flow decreased more than 10% of the former measurement (Fig. 1). Flow–time data between the starting and the ending point were fitted by using the equation:

$$Flow=a+b\times {\Delta Time}^{c},$$

where *Flow* and *Time* were described as L/s and s, respectively. The parameter *c* was defined as the flow index [14].

### Definition of low inspiratory effort

In the present study, the potential injurious low inspiratory effort was defined based on the three criteria introduced by previous studies, including (1) WOB less than 0.3 J/L [21]; (2) PTP_es_ per minute less than 50 cmH_2_O•s/min [11]; and (3) P_mus_ less than 5 cmH_2_O [15], adding “or IE index more than 10%” for all criteria [23].

### Statistical analysis

Categorical variables are expressed as counts and percentages, and continuous data are presented as median (25–75th percentile).

Pearson’s correlation was performed to evaluate the association of flow index with P_es_-derived inspiratory effort assessment parameters, including P_mus_, PTP_es_ per breath, PTP_es_ per minute, and WOB.

The incidence of low inspiratory effort was reported as percentage and 95% confidence interval (CI). The agreement of low effort identified by the three definitions was analyzed using the interclass correlation coefficient (ICC) with the two-way-random model. 95% CI of ICC was also calculated.

The primary endpoint of the present study was the validity of the flow index to detect potential injurious low inspiratory effort. The diagnostic accuracy of flow index and other non-invasive parameters (RSBI, and MV) was analyzed using the receiver operating characteristic curve (ROC), and the area under the curve (AUC) was calculated. A comparison of AUCs was performed using the DeLong test. The best cutoff value for the flow index to identify low effort was calculated using Youden’s index. For flow index, sensitivity, specificity, positive and negative predictive values (PPV and NPV), and accuracy with respective 95% CI were calculated using the best cutoff value derived from the present study and the value reported by Albani et al. [15].

Datasets with low inspiratory effort were identified according to the three definitions adopted in the present study. The association of low effort with pressure support level, type of brain injury, consciousness impairment as indicated by the Glasgow Coma Scale (GCS), and the use of analgesia and/or sedation was analyzed using a multivariate model with an enter logistic regression. Odds ratios (ORs) and 95% CIs were calculated for each factor.

The statistical analysis was conducted with SPSS 26.0 software. A *P*-value lower than 0.05 was regarded as statistically significant.

## Results

Patients’ characteristics are shown in Table 1. At a median (25–75th percentile) pressure support of 7 (6–8) cmH_2_O, the RR and V_T_ were 19 (16–22) breaths/min and 8.6 (7.4–9.8) ml/kg PBW, respectively. The three P_es_-derived parameters used to define the low inspiratory effort were WOB of 0.20 (0.12–0.57) J/L, PTP_es_ per minute of 72.6 (46.6–138.6) cmH_2_O•s/min, and P_mus_ of 5.6 (3.2–10.6) cmH_2_O. The median (25–75th percentile) of flow index was 1.7 (1.4–2.2), ranging from 1.0 to 4.7.

**Table 1:** Patients’ characteristics

Variables	*N* = 100
Male sex	67 (67)
Age (years)	53 (39–64)
Type of brain injury	
Stroke	44 (44)
Post-operation for brain tumors	37 (37)
Traumatic brain injury	19 (19)
GCS	10 (7–11)
Pressure support (cmH_2_O)	7 (6–8)
PEEP (cmH_2_O)	5 (5–8)
FiO_2_	0.4 (0.4–0.4)
RR (breaths/min)	19 (16–22)
V_T_ (ml/kg PBW)	8.6 (7.4–9.8)
MV (L/min)	10.4 (8.2–12.7)
RSBI	35 (25–47)
PaO_2_/FiO_2_	245 (198–317)
PaCO_2_ (mmHg)	37 (34–41)
∆P_es_ (cmH_2_O)	4.6 (2.7–8.3)
PEEPi (cmH_2_O)	1.2 (0.8–2.1)
P_mus_ (cmH_2_O)	5.6 (3.2–10.6)
PTP_es_ per breath (cmH_2_O•s)	3.8 (2.3–7.3)
PTP_es_ per minute (cmH_2_O•s/min)	72.6 (46.6–138.6)
WOB (J/L)	0.20 (0.12–0.57)
Flow index	1.7 (1.4–2.2)

Categorical variables are shown as number (percentage); continuous variables are shown as median (interquartile range)

*GCS* Glasgow Coma Scale, *MV* minute ventilation, *PBW* predicted body weight, *PEEP* positive end-expiratory pressure, *PEEPi* intrinsic positive end-expiratory pressure, *P*_*mus*_ inspiratory muscle pressure, *PTP*_*es*_ esophageal pressure–time product, *ΔP*_*es*_ tidal swing of esophageal pressure, *RR* respiratory rate, *RSBI* rapid shallow breathing index, *VT* tidal volume, *WOB* work of breathing

**Fig. 2:**
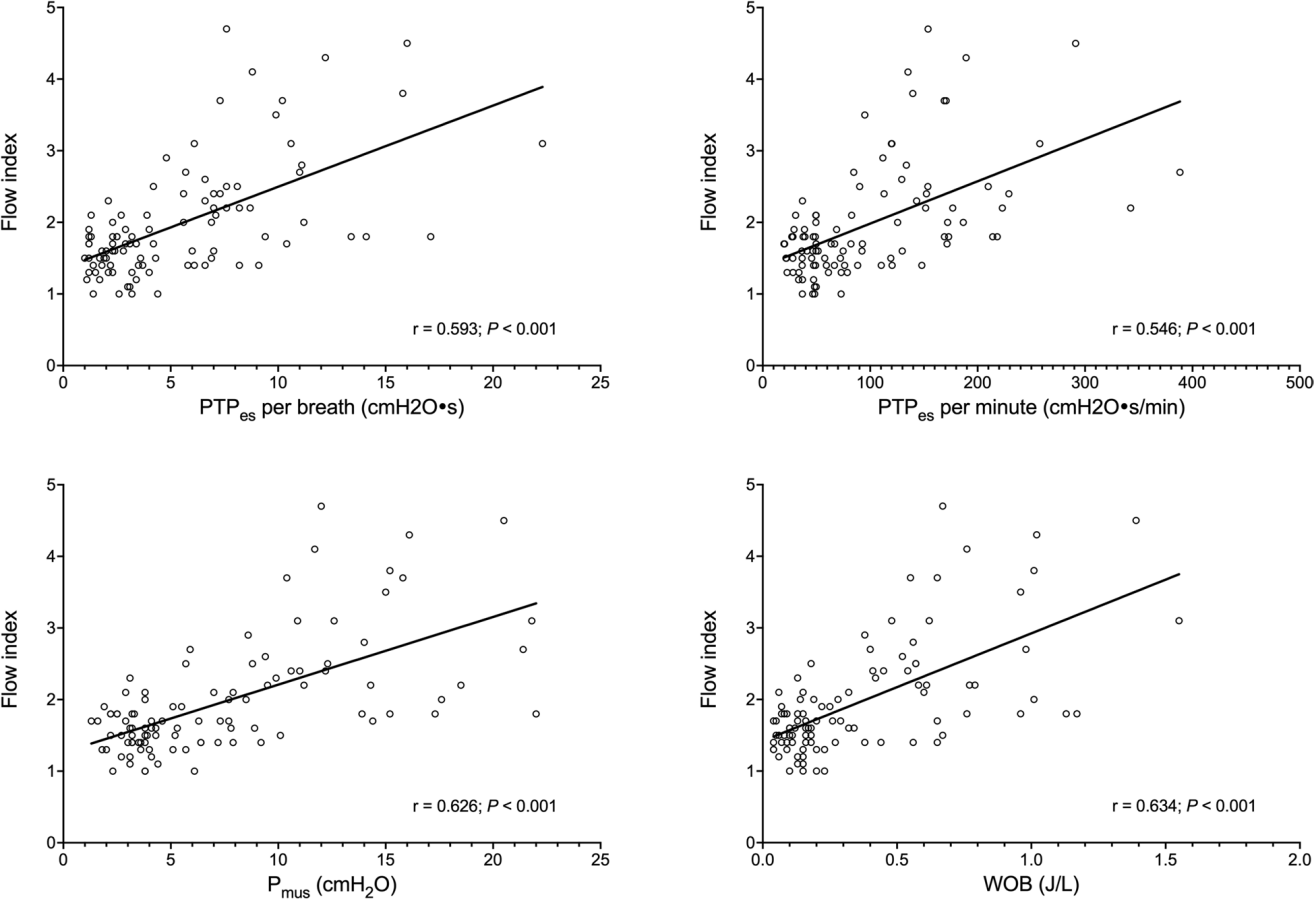
Correlation of flow index with inspiratory effort parameters derived from esophageal pressure. The flow index correlated significantly with the esophageal pressure–time product (PTP_es_) per breath and per minute, inspiratory muscle pressure (P_mus_), and work of breathing (WOB). Pearson correlation coefficient (r) is shown

Moderate correlations were found between flow index and P_es_-derived parameters for inspiratory effort evaluation, including P_mus_, PTP_es_ per breath and per minute, and WOB (Fig. 2, Pearson correlation coefficients ranged from 0.546 to 0.634, *P* < 0.001).

**Fig. 3:**
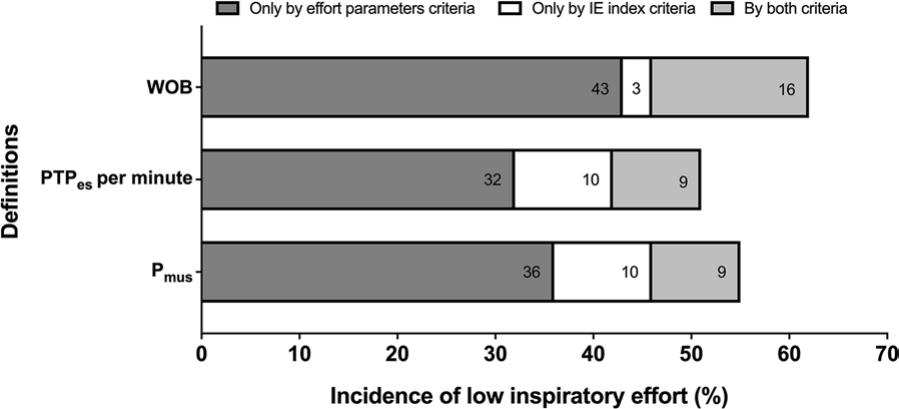
Incidence of low inspiratory effort by different definitions. Data are shown as low inspiratory effort only defined by criteria of work of breathing (WOB), esophageal pressure–time product (PTP_es_) per minute, or inspiratory muscle pressure (P_mus_), only defined by criteria of ineffective effort (IE) index, and defined by both effort parameters based on esophageal pressure and IE index criteria

Incidence (95% CI) of low inspiratory effort was 62% (51.7–71.4%), 51% (40.9–61.1%), and 55% (44.8–64.9%) by the definition of WOB, PTP_es_ per minute, and P_mus_, respectively (Fig. 3). ICC (95% CI) of agreement among the three definitions was 0.923 (0.891–0.946).

**Fig. 4:**
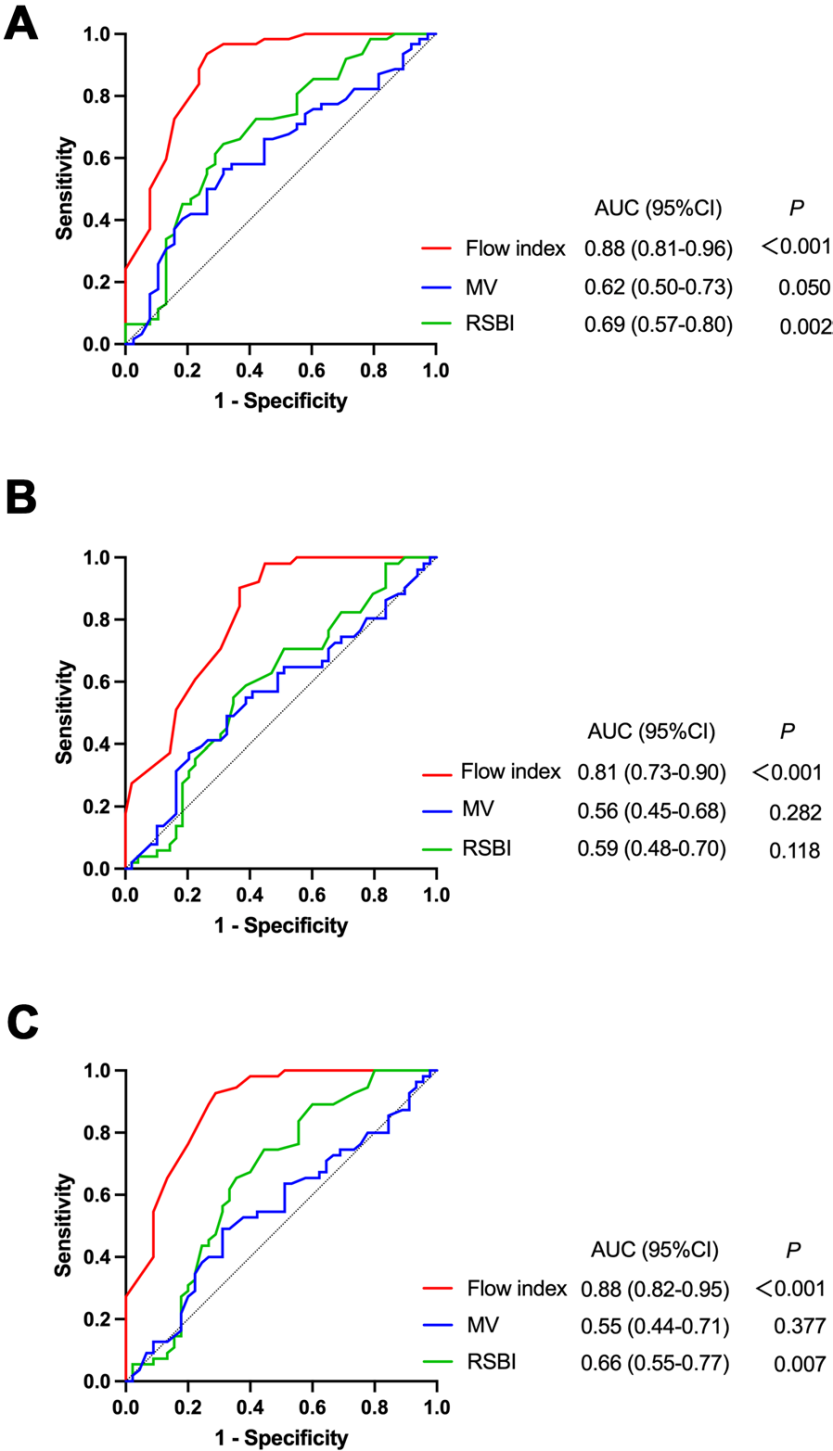
Receiver operating characteristic curve of non-invasive parameters for detecting low inspiratory effort. The area under the receiver operating characteristic curve (AUC) and 95% confidence interval (CI) are shown. By definition of work of breathing (**A**), the AUC of flow index was significantly higher than those of rapid shallow breathing index (RSBI) (*P* = 0.002) and minute ventilation (MV) (*P* < 0.001). By definition of esophageal pressure–time product per minute (**B**), the AUC of flow index was significantly higher than the other parameters (vs. RSBI: *P* = 0.002; vs. MV: *P* < 0.001). By definition of inspiratory muscle pressure (**C**), the AUC of flow index was significantly higher than the other two parameters (all *P* < 0.001)

Figure 4 shows the results of the ROC analysis for flow index and other non-invasive parameters to detect low effort. By each of the three definitions, the AUC of the flow index (0.81–0.88) was significantly higher than that of MV and RSBI (0.55–0.69) (Fig. 4). No significant differences were found in the AUCs of flow index among the three definitions (*P* > 0.05).

The cutoff value of the flow index to detect low inspiratory effort was 2.1, 2.0, and 2.0 by the definition of WOB, PTP_es_ per minute, and P_mus_, respectively. Because we considered the sensitivity of the flow index to be the most important feature to avoid missing the detection of low effort, we set 2.1 as the cutoff value for all three definitions in diagnostic performance analysis. Although specificity was relatively low (0.57–0.71), high sensitivity (0.95–0.96) and NPV (0.90–0.93) were found (Table 2). The PPVs ranged from 0.70 to 0.84. By using a higher cutoff value (2.6) previous reported to detect low inspiratory effort [15], higher sensitivity and NPV (equals to 1.00), as well as lower specificity (0.33–0.42) and PPV (0.61–0.74), were found in our cohort (Table 3).

**Table 2:** Diagnostic performance of flow index to detect low inspiratory effort using the cutoff value derived from the present study^a^

Definition	Sensitivity	Specificity	PPV	NPV	Accuracy
WOB	0.95 (0.87–0.99)	0.71 (0.54–0.85)	0.84 (0.76–0.90)	0.90 (0.75–0.97)	0.86 (0.78–0.92)
PTP_es_ per minute	0.96 (0.87–0.99)	0.57 (0.42–0.71)	0.70 (0.63–0.76)	0.93 (0.78–0.98)	0.77 (0.68–0.85)
P_mus_	0.96 (0.87–0.99)	0.62 (0.47–0.76)	0.76 (0.68–0.82)	0.93 (0.78–0.98)	0.81 (0.72–0.88)

^a^The cutoff value of flow index derived from the present study was 2.1

Between parentheses is the 95% confidence interval (95% CI)

Abbreviations: *NPV* negative predictive values, *P*_*mus*_ inspiratory muscle pressure, *PPV* positive predictive values, *PTP*_*es*_ esophageal pressure–time product, *WOB* work of breathing

**Table 3:** Diagnostic performance of flow index to detect low inspiratory effort using the cutoff value derived from the previous study^a^

Definition	Sensitivity	Specificity	PPV	NPV	Accuracy
WOB	1.00 (0.94–1.00)	0.42 (0.26–0.59)	0.74 (0.68–0.79)	1.00 (1.00–1.00)	0.78 (0.69–0.86)
PTP_es_ per minute	1.00 (0.93–1.00)	0.33 (0.20–0.48)	0.61 (0.56–0.65)	1.00 (1.00–1.00)	0.67 (0.57–0.76)
P_mus_	1.00 (0.94–1.00)	0.36 (0.22–0.51)	0.65 (0.60–0.70)	1.00 (1.00–1.00)	0.71 (0.61–0.80)

^a^The cutoff value of flow index derived from the previous study was 2.6 [14]

Between parentheses is the 95% confidence interval (95% CI)

*NPV* negative predictive values, *P*_*mus*_ inspiratory muscle pressure, *PPV* positive predictive values, *PTP*_*es*_ esophageal pressure–time product, *WOB* work of breathing

Multivariate logistic analysis showed that only the pressure support level was significantly associated with low inspiratory effort (OR ranged from 1.36 to 1.45, *P* < 0.05) (Table 4). The type of brain injury, GCS, and the use of analgesia and/or sedation did not enter the model of factors associated with low inspiratory effort.

**Table 4:** Potential factors associated with low inspiratory effort

Factors	OR (95% CI)	*P*
WOB definition		
Pressure support level	1.36 (1.07–1.79)	0.018
Type of brain injury		
Post-operation for brain tumors	1 (Reference)	
Traumatic brain injury	1.71 (0.43–7.55)	0.456
Stroke	1.06 (0.29–3.99)	0.933
GCS	1.00 (0.80–1.24)	> 0.999
Use of analgesics and/or sedatives	0.37 (0.13–1.00)	0.052
PTP_es_ per minute definition		
Pressure support level	1.50 (1.17–2.00)	0.003
Type of brain injury		
Post-operation for brain tumors	1 (Reference)	
Traumatic brain injury	1.00 (0.26–3.83)	0.997
Stroke	1.05 (0.30–3.75)	0.934
GCS	1.06 (0.86–1.32)	0.564
Use of analgesics and/or sedatives	0.57 (0.20–1.53)	0.264
P_mus_ definition		
Pressure support level	1.45 (1.13–1.92)	0.006
Type of brain injury		
Post-operation for brain tumors	1 (Reference)	
Traumatic brain injury	1.66 (0.44–6.87)	0.466
Stroke	0.98 (0.28–3.54)	0.980
GCS	1.09 (0.87–1.34)	0.453
Use of analgesics and/or sedatives	0.40 (0.14–1.06)	0.070

*CI* confidence interval, *GCS* Glasgow Coma Scale, *OR* odds ratio, *P*_*mus*_ inspiratory muscle pressure, *PTP*_*es*_ esophageal pressure–time product

## Discussion

The present analyses of previously published data in mechanically ventilated brain-injured patients show that: (1) flow index, a novel parameter of inspiratory flow–time waveform fitting, is associated with P_es_-derived inspiratory effort assessment parameters; (2) high sensitivity suggests that flow index can be used as a valid instrument to screen low inspiratory effort, and high NPV indicates a high probability of flow index to exclude cases without the condition of interest, while the relatively low specificity and PPV suggest that the flow index is less likely to rule in low effort when following values below the cutoff point; and (3) low inspiratory effort is not uncommon in brain-injured patients undergoing PSV, and the primary cause might have been over-assistance of pressure support.

The first difficulty encountered during the design of the present analysis was the definition of potential injurious low inspiratory effort. Although P_es_-based parameters have been used as the golden standard for assessing inspiratory effort [7–10], there is no consensus on the criteria for low effort in critically ill patients undergoing mechanical ventilation. Therefore, we included the three most commonly used parameters, including WOB, PTP_es_ per minute, and P_mus_, to define the low effort. These criteria are all derived from P_es_ waveform analysis which was considered the gold diagnosis. In accordance with previous studies, the lower limits of these parameters in healthy subjects at rest were selected as the criteria for definition [11, 15, 23]. And our results showed a high agreement for low effort diagnosis among these three definitions (ICC 0.923, 95% CI 0.891–0.946).

In the present study, we also included the severity of ineffective triggering in the definition of potential injurious low effort as described by Pletsch-Assuncao and coworkers [23]. The main factors associated with ineffective triggering include low respiratory drive and effort, high trigger threshold loading (PEEPi), and insensitive trigger setting [24]. In our unit, a relatively sensitive trigger setting (1–2 L/min for flow-trigger and 1.5–3 cmH_2_O for pressure-trigger) is routinely employed. Because only breaths without patient–ventilator asynchrony were included for measurements of P_es_-derived effort parameters and flow index, this resulted in a low measured PEEPi (median of 1.2 with 25–75th percentile of 0.8–2.1 cmH_2_O) in our assessments (Table 1). Notably, diagnosing low effort according to IE criteria alone was uncommon in our data, especially for the WOB definition (Fig. 3). However, further clinical outcome studies are needed to determine whether to incorporate ineffective triggering into the diagnosis of low inspiratory effort.

For bedside monitoring of inspiratory effort without additional invasive procedures, several P_aw_-based instruments have been developed, including P0.1 [11], ΔP_OCC_ [12], and PMI [13]. Studies have shown that these parameters correlate with P_es_-based effort assessing instruments and can reliably detect high and low inspiratory effort. However, these monitoring methods require airway manipulation and thus can only be performed intermittently, which may hinder their use as screening tools to detect low inspiratory effort. The newly introduced flow index, fitting the descending portion of inspiratory flow with time, represents the relationship of the patient’s inspiratory effort with ventilator flow insufflation after triggering [14]. It has been demonstrated by a single-center study that flow index correlates with inspiratory effort [14].

In Albani and coworkers’ study, pressure support was titrated to obtain low, intermediate, and high inspiratory effort, and they found the flow index was accurate to detect low inspiratory effort [15]. These results may be helpful for adjustment of pressure support to avoid over-assistance in a specific patient. In the present study, we were deliberately interested in the diagnostic performance of flow index as a screening tool to detect low inspiratory effort. ROC analysis showed that the flow index could accurately detect low effort (AUC 0.81–0.88, Fig. 4). Using less than 2.1 as the cutoff value, high sensitivity (0.95–0.96) indicated an excellent performance of the flow index as a screening tool (Table 2). Meanwhile, high NPV (0.90–0.93) indicated a high probability of excluding cases without low effort when the flow index was higher than or equal to 2.1. Similar diagnostic performances to detect low inspiratory effort were found in our cohort when using the previously reported cutoff value of 2.6 (Table 3) [15]. Additionally, we used datasets collected at clinical pressure support settings and did not perform adjustments. Therefore, our results may be more useful to screen low effort in the patient population undergoing PSV. However, it should be emphasized that lower specificity and PPV may indicate that the flow index is less likely to rule in low effort when values below the cutoff point are followed. Clinicians should use the flow index with caution when confirming the low inspiratory effort is the primary purpose.

Notably, the high inspiratory effort is also detrimental to mechanically ventilated patients. Vigorous inspiratory effort due to inadequate assistance may increase lung stress and strain, both global and regional, which is deemed as the major mechanism of patient self-inflicted lung injury [25]. Therefore, detecting high effort may be an important issue in preventing patient self-inflicted lung injury. However, our data do not allow us to describe whether the flow index can reliably detect high effort because of a relatively low incidence of high effort in the present cohort. Although Albani and coworkers demonstrated that the flow index is accurate in detecting high inspiratory effort during PSV [15], further analysis is required.

Unlike other studies on the general critically ill population [11–15], the present study analyzed brain-injured patients. The low inspiratory effort may have resulted from either low respiratory drive due to original brainstem impairment or over-assistance of mechanical ventilation, or inspiratory muscle dysfunction which is likely mainly due to over-assistance [7]. Because of the retrospective nature of the analysis, we did not have data on the prevalence and severity of brainstem damage in our cohort. Multivariate logistic analysis showed that low effort was only associated with pressure support level, but not with the type of brain injury, level of consciousness, and use of sedation/analgesia, which implied that the main reason for low effort might be over-assistance in our group of patients. However, this result should be interpreted with caution because a lot of data on brainstem impairment were not collected. Given the increased use of PSV in brain-injured patients [16, 26, 27], further studies are warranted in this population.

There are limitations in the present study. First, there is still the lack of a universally accepted definition of low inspiratory effort. We included the three most used P_es_-derived criteria plus ineffective triggering. Our results showed a high agreement for the diagnosis of low effort among these criteria. Second, we did not analyze the high inspiratory effort in the present study, mainly because high effort rarely occurred in our cohort. Third, this was a retrospective analysis of previous data in brain-injured patients. Our results may not be applicable to other populations. Fourth, we did not measure other non-invasive inspiratory effort parameters, such as P0.1, ΔP_OCC_ and PMI, because no formal airway occlusion was performed in the original research. Therefore, we cannot provide the comparison of flow index with these parameters in diagnostic performance for detecting low effort.

## Conclusions

The flow index is associated with P_es_-based inspiratory effort measurements. Flow index can be used as a valid instrument to screen low inspiratory effort with a high probability to exclude cases without the condition. Our results highlight further investigation and development of flow index as a new ventilator monitoring modality for inspiratory effort assessment.

## Data Availability

The datasets used and/or analyzed during the current study are available from the corresponding author on reasonable request.

## Abbreviations

AUC: Area under the curve
CI: Confidence interval
FiO_2_: Inspired oxygen fraction
GCS: Glasgow Coma Scale
ICC: Interclass correlation coefficient
IE: Ineffective effort
MV: Minute ventilation
NPV: Negative predictive values
OR: Odds ratio
P_aw_: Airway pressure
PBW: Predicted body weight
P_cw_: The static recoil pressure of the chest wall
PEEP: Positive end-expiratory pressure
PEEPi: Intrinsic positive end-expiratory pressure
P_es_: Esophageal pressure
PMI: Pressure muscle index
P_mus_: Inspiratory muscle pressure
PPV: Positive predictive values
PSV: Pressure support ventilation
PTP: Pressure–time product
PTP_es_: Esophageal pressure–time product
P0.1: Airway occlusion pressure
ΔP_es_: Tidal swing of esophageal pressure
∆P_OCC_: The swing in P_aw_ generated by the patient’s respiratory effort
ROC: Receiver operating characteristic curve
RR: Respiratory rate
RSBI: Rapid shallow breathing index
V_T_: Tidal volume
WOB: Work of breathing
